# Ethnoracial and rural-urban differences in female sterilization in Bolivia, Colombia, Guatemala, and Peru

**DOI:** 10.3389/fgwh.2025.1582729

**Published:** 2025-08-04

**Authors:** Lucrecia Mena-Meléndez

**Affiliations:** Department of Applied Health Science, Indiana University, Bloomington, IN, United States

**Keywords:** female sterilization, indigenous, afro-descendent, ethnoracial minorities, rural-urban inequality, Latin America

## Abstract

**Introduction:**

In Latin America, 26 percent of women use sterilization to avert unwanted pregnancies. Although sterilization provides first-tier effectiveness, long-acting continuation over time, and life-long cost-benefit, previous research has documented persistent inequalities in access and use worldwide.

**Methods:**

This study uses Demographic and Health Survey (DHS) data for Bolivia, Colombia, Guatemala, and Peru (1986-2015), to explore ethnoracial, geographic, socioeconomic, individual, and reproductive differences in female sterilization [N (level-1 women) = 112,135; N (level-2 clusters) = 4,946].

**Results:**

Results from descriptive analyses and logistic multilevel regression models indicate that ethnoracial minorities and rural women had lower odds of reporting female sterilization as their current contraceptive method. Compared to Bolivian women, Colombian and Guatemalan women had higher odds of reporting sterilization, while Peruvian women had lower odds. Older, wealthier, more educated, and those employed outside the home had higher odds of reporting sterilization. Additionally, those older at first birth, with higher parity, with a longer interval since preceding birth, and with previous experience with unintended pregnancies had higher odds of reporting sterilization.

**Discussion:**

Findings suggest that social, geographic, and structural factors may shape equitable access to sterilization for some groups. Future efforts should prioritize reducing access gaps between ethnoracial groups and rural-urban populations by strengthening health systems and ensuring culturally appropriate, equitable care.

## 1 Introduction

More than one-fourth of married or cohabiting women of reproductive age in the world—who use any contraceptive method—rely on sterilization (1). With approximately 219 million globally, this makes sterilization the most commonly used contraceptive method. Latin America and the Caribbean is a highly reliant region, with 26 percent of women of reproductive age using sterilization to avert unwanted pregnancies (1). Through either a minilaparotomy or laparoscopy procedure, female sterilization involves obstruction of the fallopian tubes (with ligatures, clips, bands, or electrocoagulation), impeding sperm transport to the tube, where fertilization of the egg occurs (2, 3). Permanent contraception (PC), such as sterilization, provides superior benefits compared to short-acting reversible contraception (SARC) and equal to and/or superior benefits to long-acting reversible contraception (LARC).^1^ First, sterilization is a first-tier highly effective method, with fewer than 1 in 100 (0.01%) women getting pregnant within 1 year of having the surgery; second, it offers long-acting continuation over time given that it is considered, for the most part, a permanent contraceptive method; third, it provides life-long cost-benefits as it involves one single procedure, which typically requires no follow-up care, and which becomes cheaper over the long-term than other methods (2, 4).

Despite this, previous research has documented persistent inequalities in sterilization access and use worldwide, particularly concerning socioeconomic status, race/ethnicity, education, geography, fertility goals and intentions, and other individual characteristics (5–8). On the one hand, some studies have found that female sterilization remains most common among socioeconomically disadvantaged and ethnoracial minorities worldwide (7–10). In the United States, researchers have documented differences in sterilization by race, with minority women more likely to undergo the procedure than non-minority women (11, 12). Other research has also looked at the geography of sterilization, finding that where someone lives may determine their sterilization outcome (13). On the one hand, research has found that rural women have an increased likelihood of undergoing sterilization compared to urban and suburban women (14), while other research has found the opposite (15).

More broadly on sexual and reproductive health in Latin America and the Caribbean, researchers have found wealth-related inequalities in the use and prevalence of LARC (16). Other research has also assessed differences in contraceptive nonuse in the region, finding that women who reported more wealth, more education, were employed in industries outside the home, were married/living together, and were older, had a lower risk of reporting contraceptive nonuse (17). Despite this, research on the use and access of sterilization in this region is much more limited. Some research in Colombia has reported observed differences in sterilization rates, with disadvantaged women, such as young, poor, and less educated, relying on sterilization more than others (18). Other research reported that public sterilization programs have used economic incentives to increase the number of sterilization procedures among Afro-Colombian women (19). However, in a multi-county research study, Ugaz et al. (20) found that female sterilization is most common among people with higher wealth, particularly in Latin America and the Caribbean.

Compared to SARC and LARC use, which are reversible methods more commonly used to decrease, delay, or space pregnancies (21), sterilization is more commonly used by women who want to prevent pregnancy permanently, given that they do not want any more children (22, 23). Arguably, in these cases, sterilization is a preferred contraceptive method to prevent childbearing because it helps overcome poor human compliance and errors that may arise with other contraceptive regimes (24). However, if sterilization inequalities exist—across socioeconomic status, ethnoracial identity, geography, or other characteristics—and some women are unable to access or use sterilization to prevent future pregnancies despite wanting to, this may signify that social, geographic, and structural determinants are impeding women's abilities to exercise their reproductive rights and control their bodies and fertility. With very limited previous research that has assessed female sterilization in Latin America, more research on this is warranted. The present study aims to fill this gap by exploring ethnoracial, geographic, socioeconomic, individual, and reproductive differences in female sterilization in Bolivia, Colombia, Guatemala, and Peru.

## 2 Material and methods

### 2.1 Data and sample

This analysis uses pooled cross-sectional Demographic and Health Survey (DHS) data from Bolivia, Colombia, Guatemala, and Peru—collected between 1986 and 2015—to conduct a population-based cross-sectional study of female sterilization. DHS data is a publicly-available, nationally-representative survey of women aged 15–49 collected by ICF International in collaboration with host country governments (25). The survey employs a two-stage stratified cluster-sampling design to randomly select women within households and clusters.^2^

For this study, country selection was guided by both theoretical and practical considerations. Theoretically, Bolivia, Colombia, Guatemala, and Peru have sizable indigenous, afro-descendent, and/or other minority populations and distinct histories of reproductive governance (26), making them relevant for understanding ethnoracial disparities in sterilization. According to their most recent national censuses, these populations represent approximately 41% of the population in Bolivia, 14% in Colombia, 44% in Guatemala, and 26% in Peru. Practically, these four countries were the only ones in Latin America with recent and multiple waves of DHS data collected between 1986 and 2015 that included both contraceptive use and measures of ethnoracial identity. Other Latin American countries with high ethnic and/or racial diversity (e.g., Brazil, Ecuador, Mexico) were excluded due to outdated surveys, the availability of only a single DHS wave, or the absence of relevant variables. Supplementary Figure S1 provides a detailed country and sample selection process. The sample was limited to women of reproductive age (15–49 years), married or cohabitating, using contraception, and with complete survey data on contraceptives and sterilization.^3^ The total study sample includes 112,135 women (level-1) and 4,946 clusters (level-2). This study was approved by the Institutional Review Board at the University of California, Los Angeles.

### 2.2 Measures

#### 2.2.1 Dependent Variable

The dependent variable in this study is female sterilization, which indicates whether a woman relies on sterilization as their contraceptive method. A dichotomous outcome was constructed using a variable that was originally categorical. Participants were asked: “Which [contraceptive] method are you using?” and provided with a list of 15 modern and traditional contraceptive methods.^4^ The responses for women who reported using sterilization as their contraceptive method were coded as one (1) and the responses for women who reported not using sterilization as their contraceptive method (using another modern or traditional contraceptive method) were coded as zero (0). Participants who reported not using contraception were excluded from the analysis. Table 1 presents percentage distributions of the prevalence of sterilization and ethnoracial self-identification by and across countries.

**Table 1:** Percentage distribution of prevalence of sterilization and ethnoracial self-identification of women (married or cohabitating, using contraception, aged 15–49) by and across countries.

	Bolivia		Colombia		Guatemala		Peru		Four Countries	
	Prevalence of sterilization		Prevalence of sterilization		Prevalence of sterilization		Prevalence of sterilization		Prevalence of sterilization	
	Yes	No	Yes	No	Yes	No	Yes	No	Yes	No
Indigenous, Afro-descendent, and/or Other minority	6.94	93.06	45.25	54.75	24.91	75.09	1.95	98.05	11.63	88.36
Non-indigenous, Afro-descendent, and/or Other minority	16.49	83.51	44.87	55.13	36.84	63.16	9.61	90.39	19.69	80.31
All	12.80	87.20	44.91	55.09	31.18	68.82	8.21	91.79	17.55	82.44
Total sample (*N*)	**16,689**		**23,619**		**9,836**		**61,991**		**112,135**	

Bold figures represent the total samples for each one of the countries and the four countries combined.

Note: The number of cases is weighted and may not add to the total because of rounding.

Source: author's calculations of Demographic and Health Surveys data, 1986–2015.

#### 2.2.3 Independent variables

The primary independent variable in this study is ethnoracial self-identification, operationalized as a dichotomous variable indicating whether a woman self-identifies as indigenous, afro-descendent, and/or other minority or not. Self-identified ethnicity (Colombia: Indigenous, Gypsy/Rom, Raizal, Palenquero, Black/Mulatto/Afro-Colombian/Afro-descendant; Guatemala: Maya, Garifuna, Xinca, Other) and language spoken at home (Bolivia: Quechua, Aymara, Guaraní, Other; Peru: Quechua, Aymara, Other) was used to construct this measure. While DHS datasets provide ethnoracial indicators, the structure and categories vary by country and survey year. To enable cross-national comparison and maintain analytic consistency, these categories were harmonized into a binary classification: indigenous, afro-descendent, and/or other minority (1) compared to non-indigenous, afro-descendent, and/or other minority (0). This decision was informed by limitations in available categories across surveys, small sample sizes in some subgroups, and inconsistencies in coding over time. However, this dichotomization may obscure important intra-group variation and reduce cultural specificity. These trade-offs are acknowledged as a limitation, particularly in the interpretation of cross-country differences. Self-identified ethnicity and language are standard measures of ethnoracial identity used in previous research (27–29). While other research recommends using multiple self-identification measures, interviewer-ascribed phenotypic classifications, and multiple sub-categories of race and ethnicity (30, 31), DHS data does not consistently collect such measures across countries, or has only started collecting such data. Supplementary Table S1 presents percentage distributions of ethnoracial self-identification by country. Supplementary Table S2 compares how DHS survey questionnaires ask about ethnoracial self-identification in these four countries.

This study also controlled for place of residence (rural, urban), household wealth (poorest, poorer, middle, richer, and richest), years of education (0, 1–3, 4–6, 7–9, ≥10 years), occupation (not working, managerial, clerical, sales, agricultural, domestic and other services, manual labor), husband's education (0–23), age (15–19, 20–24, 25–34, 35–49), age at-first-birth (8–14, 15–19, 20–34, 35+), number of living children (1–2, 3–4, 5+), interval since preceding birth (>2, 2–4, 4+), intention of last pregnancy (intended, unintended), and previous abortion (yes, no). Table 2 presents percentage distributions of selected characteristics by ethnoracial self-identification and the entire sample.

**Table 2:** Sample characteristics of women (married or cohabitating, using contraception, aged 15–49), by ethnoracial self-identification.

Characteristic	Indigenous, Afro-descendent, and/or other minority	Non-Indigenous, Afro-descendent, and/or other minority	All
Geographic factors			
Place of residence***			
Rural	69.60	42.73	49.87
Urban	30.40	57.27	50.13
Socioeconomic factors			
Household wealth***			
Poorest	38.00	22.39	26.53
Poorer	31.83	24.72	26.61
Middle	16.34	23.25	21.42
Richer	9.34	16.97	14.94
Richest	4.49	12.67	10.50
Years of education***			
0 years	22.55	7.60	11.57
1–3 years	31.68	18.57	22.05
4–6 years	29.03	28.40	28.57
7–9 years	7.41	13.74	12.06
10+ years	9.32	31.69	25.75
Occupation***			
Not working	28.85	31.67	30.93
Managerial	2.14	4.71	4.03
Clerical	0.51	2.18	1.74
Sales	13.73	18.63	17.33
Agricultural	41.01	20.85	26.20
Domestic and services	5.35	14.84	12.32
Manual	8.40	7.12	7.46
Husband's education***	5.94	8.08	7.51
Individual and reproductive factors			
Age***			
15–19 years	0.51	0.54	0.53
20–24 years	5.78	7.40	6.97
25–34 years	41.69	45.47	44.47
35–49 years	52.02	46.59	48.03
Age at first birth*			
8–14 years	3.76	3.75	3.75
15–19 years	57.61	54.27	55.16
20–34 years	38.47	41.75	40.88
35+ years	0.16	0.23	0.21
Number of living children***			
0	0.01	0.04	0.03
1–2	10.66	20.10	17.60
3–4	33.40	43.02	40.47
≥5	55.93	36.83	41.91
Interval since preceding birth***			
>2 years	31.06	25.25	26.79
2–4 years	49.60	40.31	42.78
4+ years	19.34	34.44	30.43
Intention of last pregnancy***			
Intended	29.84	36.02	34.38
Unintended	70.16	63.98	65.62
Previous abortion***			
Yes	80.70	75.78	77.08
No	19.30	24.22	22.92
Total sample (*N*)	**26,458**	**85,677**	**112,135**

Notes: The number of cases is weighted and may not add to the total because of rounding.

To assess the significance across ethnoracial self-identification, chi-squared and t-tests were conducted.

Source: author's calculations of Demographic and Health Surveys data on 4 countries, 1986–2015; *N* = 112,135.

*p < 0.05, **p < 0.01, ***p < 0.001, ****p < 0.10.

### 2.3 Analyses

To explore the relationship between ethnoracial self-identification and female sterilization across pooled waves and countries, DHS recommendations (32) were followed to de-normalize the women's individual standard weight (for each wave and each country) by dividing the women's individual standard weight (v005) by 1,000,000. Then, a new sample weight was created by multiplying the de-normalized weight by the estimated population of women aged 15–49 in the country during the year of the DHS survey (as reported by the World Bank Open Data), and then dividing by the number of women aged 15–49 interviewed in that survey. This step adjusts for differences in survey sample size and ensures that each country contributes proportionally to the pooled analysis. This approach follows guidance outlined in DHS Analytical Reports and comparative survey methodology documentation. After weight construction, all datasets were appended, and survey design variables were specified using the *svyset* command in Stata 16 (33). Primary sampling units (v021), stratification [region (v024), urban/rural (v025)], and adjusted weights were specified to account for the complex survey design and to produce valid standard errors. All descriptive statistics and multilevel analyses were weighted accordingly. Analyses were conducted in three steps. First, the distribution of the characteristics described above was assessed across ethnoracial self-identification. Second, chi-square tests were conducted to examine bivariate associations. Third, three multilevel logistic regression models were constructed to measure the relative odds of female sterilization by ethnoracial self-identification, while controlling for selected characteristics. To respect the hierarchical design of DHS data, a two-level multilevel logistic regression approach was used, whereby individual women units (level-1) were nested within survey cluster units (level-2).^5^ The models include a random intercept at the cluster-level—to capture unobserved heterogeneity among clusters—and fixed effects for all other individual-level coefficients. Finally, survey year and country fixed effects were included in all models to account for temporal and contextual variation. Model 1 regressed female sterilization on survey year, ethnoracial self-identification, and geographic factors. Model 2 controlled for socioeconomic factors. Finally, Model 3 controlled for additional individual and reproductive factors.

Across all models, an interaction term was also included between ethnoracial self-identification and place of residence to examine the extent that place of residence moderated the relationship between ethnoracial self-identification and sterilization. Previous research indicates that in non-linear models, the product of the interaction (i.e., coefficient) can be misleading and insufficient for drawing conclusions (34–36). Based on methodological recommendations, the *margins* command in Stata was used to calculate the predicted probabilities (from the coefficient estimates in Model 3) and relied on tests of the predictive probabilities to determine whether an interactive effect existed between ethnoracial self-identification and place of residence. Supplementary File S1 presents annotated Stata code used for weight construction, model estimation, and marginal predictions. Additional code or details are available upon request. Unless noted otherwise, any differences mentioned in the following sections are statistically significant.

## 3 Results

### 3.1 Multivariate results for female sterilization by ethnoracial, geographic, socioeconomic, individual, and reproductive factors

Table 3 presents the results of the multilevel logistic models predicting female sterilization of married or cohabitating women aged 15–49 [N (level-1) = 112,135; N (level-2) = 4,946]. Results from Model 1 indicate that indigenous, afro-descendent, and/or other minority women had significantly lower odds of reporting female sterilization as their current contraceptive method compared to non-indigenous, afro-descendent, and/or other minority women (OR = 0.56, *p* < 0.001). In addition, the odds of reporting sterilization varied by geographic factors. Colombian and Guatemalan women had higher odds of reporting sterilization than Bolivian women (OR = 4.21, *p* < 0.001; OR = 2.47, *p* < 0.001, respectively), whereas Peruvian women had lower odds (OR = 0.50, *p* < 0.001). Additionally, rural women had significantly lower odds of reporting sterilization compared to urban women (OR = 0.46, *p* < 0.001).

**Table 3:** Odds ratios from multilevel logistic regression analysis predicting sterilization of women (married or cohabitating, using contraception, aged 15–49).

	Model 1				Model 2				Model 3			
Variables	Coefficient	95% C.I.	95% C.I.	S.D.	Coefficient	95% C.I.	95% C.I.	S.D.	Coefficient	95% C.I.	95% C.I.	S.D.
		Low	High			Low	High			Low	High	
Year	**1**.05***	1.03	1.08	0.01	**1**.06***	1.03	1.08	0.01	**1**.07***	1.04	1.09	0.01
Ethnoracial self-identification												
Indigenous, afro-descendent, and/or other minority (ref. = Non-indigenous, afro-descendent, and/or other minority)	**0**.56***	0.49	0.65	0.04	**0**.64***	0.55	0.74	0.05	**0**.61***	0.52	0.72	0.05
Geographic factors												
Country (ref. = Bolivia)												
Colombia	**4**.21***	3.55	4.98	0.36	**4**.34***	3.62	5.21	0.40	**5**.58***	4.66	6.67	0.51
Guatemala	**2**.47***	1.92	3.19	0.32	**2**.00***	1.54	2.60	0.27	**2**.44***	1.85	3.22	0.35
Peru	**0**.50***	0.41	0.60	0.05	**0**.51***	0.42	0.62	0.05	**0**.50***	0.41	0.61	0.05
Place of residence (ref. = Urban)												
Rural	**0**.46***	0.41	0.52	0.03	**0**.77***	0.66	0.90	0.06	**0**.77***	0.66	0.90	0.06
Indigenous, afro-descendent, and/or other minority x Rural (ref. = Non- × Urban)												
Non-indigenous, afro-descendent, and/or other minority × Rural	**0**.47***	0.42	0.53	0.03	**0**.80**	0.68	0.94	0.06	**0**.79**	0.67	0.94	0.07
Indigenous, afro-descendent, and/or other minority × Urban	**0**.59***	0.47	0.74	0.07	**0**.69***	0.55	0.86	0.08	**0**.66***	0.53	0.83	0.08
Indigenous, afro-descendent, and/or other minority × Rural	**0**.26***	0.21	0.31	0.02	**0**.47***	0.38	0.60	0.06	**0**.45***	0.36	0.58	0.06
Socioeconomic factors												
Household wealth (ref. = Poorest)												
Poorer					**1**.24***	1.07	1.44	0.09	**1**.30***	1.11	1.52	0.10
Middle					**1**.47***	1.21	1.77	0.14	**1**.57***	1.29	1.92	0.16
Richer					**1**.69***	1.36	2.10	0.19	**1**.86***	1.49	2.33	0.21
Richest					**2**.23***	1.74	2.87	0.29	**2**.36***	1.82	3.07	0.32
Years of education (ref. = 0 years)												
1–3 years					0.89	0.70	1.13	0.11	0.96	0.75	1.22	0.12
4–6 years					0.86	0.68	1.08	0.10	1.06	0.83	1.36	0.13
7–9 years					0.86	0.67	1.10	0.11	1.24	0.95	1.63	0.17
10+ years					1.05	0.81	1.37	0.14	**1**.66***	1.24	2.21	0.24
Occupation (ref. = Not working)												
Managerial					1.17	0.91	1.50	0.15	1.10	0.85	1.42	0.14
Clerical					1.06	0.77	1.44	0.17	1.11	0.81	1.52	0.18
Sales					0.91	0.78	1.08	0.08	**0**.86****	0.73	1.01	0.07
Agricultural					**0**.57***	0.46	0.71	0.06	**0**.54***	0.43	0.67	0.06
Domestic and services					1.04	0.87	1.25	0.10	0.97	0.81	1.16	0.09
Manual					1.01	0.81	1.27	0.12	0.92	0.73	1.15	0.11
Husband's education									1.01	0.99	1.02	0.01
Individual and reproductive factors												
Age (ref. = 15–19 years)												
20–24 years									**1**.62****	0.96	2.73	0.43
25–34 years									**2**.71***	1.63	4.51	0.71
35–49 years									**3**.87***	2.28	6.54	1.04
Age at first birth (ref. = 8–14 years)												
15–19 years									0.99	0.73	1.33	0.15
20–34 years									1.00	0.73	1.37	0.16
35+ years									**9**.16***	4.31	19.44	3.52
Number of living children (ref. = 1–2)												
3–4									**2**.66***	2.36	2.99	0.16
5+									**3**.14***	2.59	3.81	0.31
Interval since preceding birth (ref. ≥ 2 years)												
2–4 years									0.96	0.89	1.04	0.04
4+ years									**0**.91**	0.84	0.98	0.04
Intention of last pregnancy									**1**.23***	1.10	1.37	0.07
Previous abortion									1.09	0.96	1.23	0.07
Random effect (cluster–level)												
**N (level-1)**	**112,135**				**112,135**				**112,135**			
**N (level-2)**	**4,946**				**4,946**				**4,946**			

Notes: Statistically significant coefficients at *p* < 0.05 are bolded. The reference category is given in parentheses.

Source: author's calculations of Demographic and Health Surveys data on 4 countries, 1986–2015; N (level-1) = 112,135; N (level-2) = 4,946.

* *p* < 0.05, **p < 0.01, ***p < 0.001, ****p < 0.10.

Even after controlling for socioeconomic factors, results from Model 2 indicate that ethnoracial self-identification continues to be associated with lower odds of reporting female sterilization as a current contraceptive method (OR = 0.64, *p* < 0.001). Additionally, women who reported higher wealth—richest (OR = 2.23, *p* < 0.001)—had higher odds of reporting sterilization. Conversely, women who reported living in rural areas (OR = 0.77, *p* < 0.001) and working in agriculture (OR = 0.57, *p* < 0.001) had lower odds of reporting sterilization.

Model 3 presents the fully adjusted model after controlling for all remaining individual and reproductive factors. Once again, ethnoracial self-identification was associated with significantly lower odds of reporting female sterilization as their current contraceptive method (OR = 0.61, *p* < 0.001). Additionally, results indicate that older women—35–49 years (OR = 3.87, *p* < 0.001); older at first-birth—35 + years (OR = 9.16, *p* < 0.001); with 3–4 and 5+ living children (OR = 2.66, *p* < 0.001; OR = 3.14, *p* < 0.001); and with previous experiences with unintended pregnancies (OR = 1.23, *p* < 0.001) had higher odds of reporting sterilization. On the other hand, women who reported living in rural areas (OR = 0.77, *p* < 0.001), working in agriculture (OR = 0.54, *p* < 0.001), and with a longer interval since preceding birth—4 + years (OR = 0.91, *p* < 0.01)—had lower odds of reporting sterilization.

**Figure 1:**
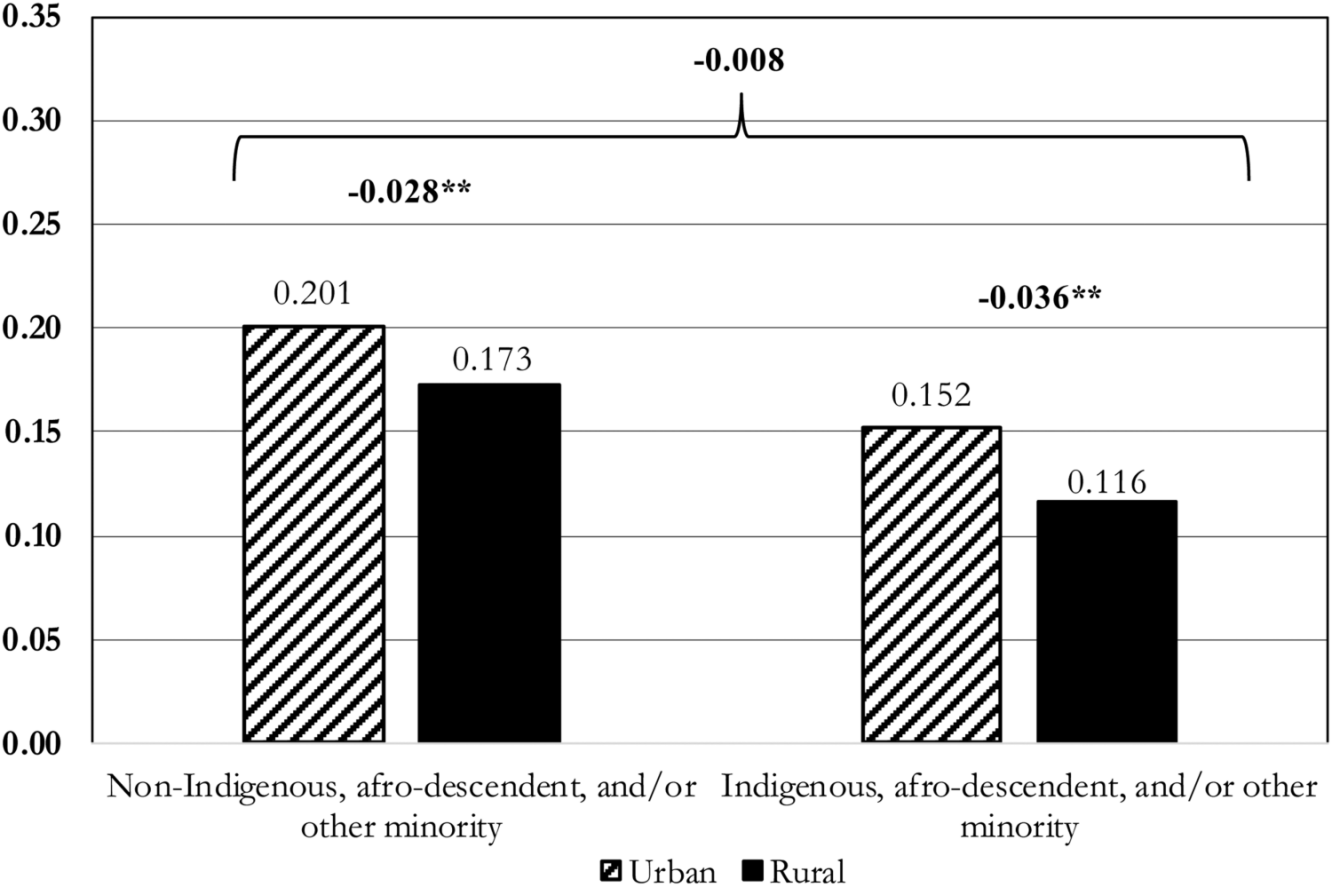
Probabilities of sterilization of women (married or cohabitating, using contraception, aged 15–49), by ethnoracial self-identification and place of residence. Source: author's calculations of Demographic and Health Surveys data on 4 countries, 1986-2015; N (level-1) = 12,135; N (level-2) = 4,946. Notes: Predicted probabilities and 95% confidence intervals are based on margins computed from the fully adjusted multilevel logistic regression (Model 3). Estimates are as follows:
•Non-Indigenous, afro-descendent, and/or other minority, Urban: 0.201 [95% CI: 0.187, 0.214]•Non-Indigenous, afro-descendent, and/or other minority, Rural: 0.173 [95% CI: 0.160, 0.185]•Indigenous, afro-descendent, and/or other minority, Urban: 0.152 [95% CI: 0.131, 0.174]•Indigenous, afro-descendent, and/or other minority, Rural: 0.116 [95% CI: 0.099, 0.132]

To properly examine the interaction between ethnoracial self-identification and place of residence on female sterilization, predicted probabilities were calculated from the fully adjusted multilevel logistic regression model (Table 3, Model 3) using the *margins* command in Stata and relying on tests of the predictive probabilities. Figure 1 presents the predicted probabilities with 95% confidence intervals and the tests of first and second differences. The first differences capture the effect of place of residence given respondents’ ethnoracial self-identification. The second difference test whether the effect of place of residence significantly differs by respondents’ ethnoracial self-identification Results indicate that among non-indigenous, afro-descendent, and/or other minority respondents, those living in rural areas had a lower predicted probability of reporting sterilization as their current contraceptive method (Pr = 0.173, 95% CI: 0.160–0.185) compared to those in urban areas (Pr = 0.201, 95% CI: 0.187–0.214; *Δ* = −0.028, *p* < 0.01). Similarly, among indigenous, afro-descendent, and/or other minority respondents, those living in rural areas had a lower probability of reporting sterilization as their current contraceptive method (Pr = 0.116, 95% CI: 0.099–0.132) than those in urban areas (Pr = 0.152, 95% CI: 0.131–0.174; *Δ* = −0.036, *p* < 0.01). The test of second difference was not statistically significant (*Δ* = −0.008, *p* > 0.05), indicating that the effect of place of residence does not vary significantly by respondents’ ethnoracial self-identification. In other words, women in urban areas had a higher probability of reporting sterilization as their current contraceptive method, regardless of ethnoracial self-identification.

## 4 Discussion

The present study used Demographic and Health Survey (DHS) data (1986–2015) to explore ethnoracial, geographic, socioeconomic, individual, and reproductive differences in female sterilization in Bolivia, Colombia, Guatemala, and Peru. Very few studies to date have examined female sterilization in this region. Four key findings emerged from this study.

First, even after controlling for all factors, ethnoracial minority women have lower odds of reporting female sterilization as their current contraceptive method compared to non-ethnoracial minority women. This finding conflicts with research in the United States that reports that minority women are more likely to undergo sterilization than non-minority women (11, 12). However, the experiences of ethnic-minority women in Latin America are substantially different from those of women in the United States, or to that end, from those of women in other parts of the world, so we should be careful when comparing across regions. In Latin America, most minorities live in extreme poverty, have high illiteracy rates, work in informal occupations, have worse health outcomes, and have limited to no access to insurance or healthcare systems (37–39). Previous research in the United States and Latin America has found that insurance and socioeconomic status are strong predictors of female sterilization (40–42). Thus, lack of insurance and/or limited access to healthcare may provide one possible explanation of why ethnoracial minority women have lower odds of reporting sterilization than non-ethnoracial minorities. This finding can also be understood through the lens of reproductive justice, which emphasizes the right to have children, not have children, and to parent in safe and sustainable communities (43). Lower odds of reporting sterilization may reflect not only limited access to insurance or healthcare systems, but also institutional discrimination—defined as the systemic exclusion of marginalized groups from reproductive autonomy through policy, provider bias, and health system neglect (44, 45). In Latin America, the intersection of poverty, rural residence, and minoritized status may restrict the conditions under which women can make fully informed and supported contraceptive choices (46).

Second, results from this study indicate that female sterilization differs across geographical factors. More generally, rural women are less likely to report sterilization. Previous research in this area is conflicting, but this finding does align with research from Latin America and other developing regions (15, 47). Generally, urban women have better sexual and reproductive health outcomes than rural women because urban areas have greater, expanded, and improved access to, delivery of, and funding for modern family planning methods and services (48, 49). In addition, it is possible that higher sterilization in urban areas is explained by normative, socio-demographic, and cultural differences between rural-urban women. For example, the desire for smaller families, higher costs of childbearing, or changes in gender and family expectations and dynamics in urban areas. Findings also indicate differences by country, with Colombian and Guatemalan women more likely to report sterilization compared to Bolivian women, while Peruvian women are less likely. These cross-country variations are reported widely in the literature and may be explained by the history of sterilization availability, promotion, and acceptance in a given country (50, 51). Despite sharing close geographic proximity, as well as centuries of ethnolinguistic, geopolitical, and historically communal legacies (52), each one of these countries is also unique, so these differences are to be expected.

Third, the findings from this study indicate significant differences in sterilization by socioeconomic factors, particularly wealth, education, and occupation. While some research has found that female sterilization remains most common among socioeconomically disadvantaged women (7), some research in Latin America has found the opposite—that female sterilization is most common among wealthier women (20, 41). Given that many countries’ public health systems do not cover the procedure, or require extensive wait times or paperwork (41), women with more wealth or education, who may also be able to afford private insurance, may have more access to information about sterilization and have the financial opportunity to undergo the procedure itself.

Fourth, findings also suggest disparities by individual and reproductive factors, particularly age, age at first birth, parity, interval since preceding birth, and previous unintended pregnancies. That is, results indicate that those who had achieved their desired fertility were more likely to report sterilization. These findings are consistent with previous research that has found that sterilization prevalence increases with age, parity, and prior experience with an unintended pregnancy (50, 53, 54). Other research has also found that providers assess risk factors for sterilization regret and adjust their advice or recommendation for the procedure depending on a patient's age, parity, and spousal agreement (55). Some research has found evidence that women have been dissuaded or refused from undergoing the procedure due to their age or parity, due to possible procedural regret (56). Given the present findings, it is possible that younger women, younger mothers, and those with fewer children, but who do not want more children, are not receiving information or advice about sterilization as a possible permanent contraceptive option, which may be compromising their reproductive autonomy. Future research could incorporate in-depth interviews to explore further how institutional, structural, and cultural factors shape women's access to sterilization in different contexts. This includes investigating the roles of consent protocols, provider bias, and localized social norms, especially for ethnoracial minority and rural women. Longitudinal or mixed-methods studies could also help assess how access and decision-making around sterilization evolve over time, particularly as health systems and policy landscapes shift. Attention to intersecting inequalities—such as geography, race/ethnicity, and insurance status—can further illuminate the social determinants of sterilization access and unmet need across Latin America.

### 4.1 Limitations

Although this study has made a substantial set of contributions, the following limitations and the need for future research should be acknowledged. First, the analyses relied on self-reported data, so the results depend on the information that respondents selectively chose to share and/or failed to recall about current or previous experiences. Particularly, the measure for ethnicity and/or race relied exclusively on self-reported ethnicity or language, which may be a conservative measure given that some individuals may not speak traditional ethnic languages or dialects, or may identify with an ethnic minority but may choose not to self-identify because of stigma and discrimination against minority identities or languages (37). Ideally, future research should use multiple measures or dimensions of race and ethnicity, including measures that account for phenotypic classifications and classifications made by others (31).

In addition, the harmonization of ethnoracial self-identification into a binary classification—indigenous, afro-descendent, and/or other minority vs. non-indigenous, afro-descendent, and/or other minority—represents a trade-off between analytical consistency and cultural specificity. While necessary for cross-national comparison, this simplification may obscure meaningful intra-group variation and reduce the precision of interpreting results across countries. These limitations are particularly relevant given the country-specific differences in how DHS surveys measure and categorize ethnoracial identity.

Second, to make comparable analytical variables across countries and waves, categorical responses were collapsed, which may have led to the loss of significant information. Additionally, pooling data across countries and waves may have artificially inflated the effective sample size, potentially resulting in overestimated statistical power and underestimated standard errors due to design effects and clustering (57). However, pooling datasets together also decreases errors from interviewer noise, poorly worded questions, data entry mistakes, and sampling variability (58). To further address this concern, recommendations from DHS (32) were followed to de-normalize the standard weight to ensure the appropriate contribution of data within each wave and country. Third, this study is unable to control for country- and period-specific characteristics not collected by the DHS (e.g., ethnoracial discrimination, religion, religious beliefs, implementation of consent procedures). Finally, this analysis is limited to four countries in Latin America, so these results cannot be blindly generalized.

## 5 Conclusion

This study used Demographic and Health Survey (DHS) data for Bolivia, Colombia, Guatemala, and Peru (1986–2015) to examine ethnoracial, geographic, socioeconomic, individual, and reproductive differences in female sterilization. Results indicate consistent differences in female sterilization across these characteristics. These disparities suggest that some groups—particularly ethnoracial minority women, rural residents, and those with fewer resources—may face structural barriers to sterilization as a contraceptive method. There is an urgent need to examine how social, geographic, and institutional factors shape who is offered, informed about, or able to obtain sterilization. Improving equity in family planning will require programs that are attentive to group-specific barriers and that promote informed, voluntary contraceptive use across diverse populations. Future efforts should prioritize reducing access gaps between ethnoracial groups and rural-urban populations by strengthening health systems and ensuring culturally appropriate, equitable care.

## Data Availability

The original contributions presented in the study are included in the article/Supplementary Material, further inquiries can be directed to the corresponding author.

## Footnotes

^1^SARCs include oral pills (combination or progestin-only), injectable, transdermal patch, vaginal ring, male condom, female condom, diaphragm, sponge, cervical cap, and spermicides and gels; LARCs include intrauterine devices and implants.

^2^DHS data is collected as follows: First, each province or region of a country is divided into small census Enumeration Areas (EAs). Using probability proportional to size (PPS), some of the areas are then selected randomly to be representative of the region or province, or their urban and rural parts, and are the primary sampling units (PSUs). The PSUs form the survey cluster units (level-2). Within each PSU, about 25-30 households are randomly selected, and all women of reproductive age in the households are interviewed. Women represent the individual women units (level-1) (26).

^3^As per DHS reporting, this study’s sample included very minimal missing data (coded as values that are missing because of nonresponse or invalid codes assigned by DHS as 96, 97, 98, or 99). For the variables included in the analysis, the ones with missing data were self-reported ethnicity (total missing=0.01%), occupation (total missing=0.07%), husband’s education (total missing=1.43%), age (total missing=0.01%), and pregnancy intention (total missing=0.07%). While minimal, any observations with missing data were excluded from this analysis.

^4^Modern methods: the pill, IUD, injection, diaphragm, NorplantTM or implant, condom, female condom, foam and jelly, female sterilization, male sterilization, other contraceptive methods, and country-specific contraceptive methods. Traditional or folk methods: lactational amenorrhea, periodic abstinence (rhythm), and withdrawal.

^5^Compared with single-level regression analysis that assumes that all individuals are independent, this methodology accounts for the fact that individuals in the same cluster may have similar characteristics (33).

## Supplementary material

Supplementary Data Sheet 1 Supplementary STATA Code.
