# Supplementary material for: Ethnoracial and rural-urban differences in female sterilization in Bolivia, Colombia, Guatemala, and Peru
Source: Front Glob Womens Health. 2025 Aug 4;6:1582729. doi: 10.3389/fgwh.2025.1582729 (PMC12358441; doi:10.3389/fgwh.2025.1582729)
Supplement: Supplementary Data Sheet 1 — Supplementary STATA Code. [file Datasheet1.pdf]

```
**********************************************************************
 Supplementary Do-File for: Ethnoracial and Rural-Urban Differences in
                            Female Sterilization in Bolivia, Colombia, Guatemala, and Peru
 Author: Lucrecia Mena-Meléndez, Ph.D. (lmenamel@iu.edu)
 Description: Core code for weight construction, multilevel model,
              and predicted probabilities for interaction analysis.
              Based on DHS data from Bolivia, Colombia, Guatemala, Peru (1986–2015).
 Note: This code assumes data cleaning, weight denormalization, and harmonization
        across countries and waves are complete. The provided script reproduces
        the main statistical analysis reported in the manuscript, including the
        model used in Table 3 and the interaction estimates visualized in Figure 1.

**********************************************************************

*** Step 1: Generate weight, strata, and specify complex survey design
* Generate weight
generate weight = v005_denorm/1000000
* Generate strata
egen strata = group(v024 v025), label
* Specify complex survey design
svyset [pweight=weight], psu(v021) strata(strata)

*** Step 2: Run multilevel logistic regression with random intercept
* Model 1
melogit sterilized_women year i.country ib0.indigenous#ib0.region if sample_sterilization==1 & married_cohab==1 [pw=weight] || v001:, or
* Model 2
melogit sterilized_women year i.country ib0.indigenous#ib0.region i.household_wealth ib0.years_educ i.respondent_occupation if sample_sterilization==1 & married_cohab==1 [pw=weight] || v001:, or
* Model 3
melogit sterilized_women year i.country ib0.indigenous#ib0.region i.household_wealth ib0.years_educ i.respondent_occupation husbands_educ ib1.age_four_groups i.age_first_birth ib1.living_children i.birth_interval i.pregnancy_intention i.abortion if sample_sterilization==1 & married_cohab==1 [pw=weight] || v001:, or
est store sterilized_women_mod

*** Step 3: Predicted probabilities by ethnoracial identity and place of residence
melogit sterilized_women year i.country ib0.indigenous ib0.region i.household_wealth ib0.years_educ i.respondent_occupation husbands_educ ib1.age_four_groups i.age_first_birth ib1.living_children i.birth_interval i.pregnancy_intention i.abortion if sample_sterilization==1 & married_cohab==1 [pw=weight] || v001:, or
mtable, at(indigenous=(0 1) region=(0 1)) post
* Effect of living in a rural area for non-indigenous, afro-descendent, other minority
mlincom 2 - 1, stat(est se p)
* Effect of living in a rural area for indigenous, afro-descendent, other minority
mlincom 4 - 3, stat(est se p)
* Second differences
mlincom (4-3) - (2-1), stat(est se p)

* End of Supplementary Do-File
```
