# Supplementary material for: Ethnoracial and rural-urban differences in female sterilization in Bolivia, Colombia, Guatemala, and Peru
Source: Front Glob Womens Health. 2025 Aug 4;6:1582729. doi: 10.3389/fgwh.2025.1582729 (PMC12358441; doi:10.3389/fgwh.2025.1582729)
Supplement: Supplementary file 2 [file Datasheet2.docx]

Supplementary Material

# Supplementary Figures and Tables

## Supplementary Tables

### Table 1

| **Supplementary Table 1** | | | | |
| --- | --- | --- | --- | --- |
| **Percentage distribution of ethnoracial self-identification of women (married or cohabitating, using contraception, aged 15–49)** | | | | |
| **(Source: author's calculations of Demographic and Health Surveys data on 4 countries, 1986–2015; N=112,135)** | | | | |
|  |  |  |  | **Total sample (N)** |
| **Bolivia** | Indigenous, Afro-descendent, and/or Other Minority | Indigenous | 38.60 | **5,206** |
|  |  | Afro-descendent | — |  |
|  |  | Other minority | 0.90 |  |
|  | Non-indigenous, Afro-descendent, and/or Other minority |  | 60.50 | **11,483** |
| **Colombia** | Indigenous, Afro-descendent, and/or Other Minority | Indigenous | 3.32 | **3,674** |
|  |  | Afro-descendent | 7.14 |  |
|  |  | Other minority | 0.02 |  |
|  | Non-indigenous, Afro-descendent, and/or Other minority |  | 89.52 | **19,945** |
| **Guatemala** | Indigenous, Afro-descendent, and/or Other Minority | Indigenous | 47.24 | **4,384** |
|  |  | Afro-descendent | 0.09 |  |
|  |  | Other minority | 0.11 |  |
|  | Non-indigenous, Afro-descendent, and/or Other minority |  | 52.56 | **5,452** |
| **Peru** | Indigenous, Afro-descendent, and/or Other Minority | Indigenous | 18.21 | **13,194** |
|  |  | Afro-descendent | — |  |
|  |  | Other minority | 0.04 |  |
|  | Non-indigenous, Afro-descendent, and/or Other minority |  | 81.75 | **48,797** |
| Note: The number of cases is weighted and may not add to the total because of rounding. | | |  |  |

### Table 2

| **Supplementary Table 2** | | | | |
| --- | --- | --- | --- | --- |
| **Survey question (“v131”) on ethnicity (family language)** | | | | |
| **(Source: Demographic and Health Surveys survey questionnaires for Bolivia, Colombia, Guatemala, and Peru, 1986–2015)** | | | | |
|  | **Bolivia** | **Colombia** | **Guatemala** | **Peru** |
| **Survey question (Spanish)** | ¿Qué idiomas o lenguas habla? | De acuerdo con su cultura, pueblo o rasgos físicos, es o se reconoce como: | Usted ¿cómo se considera: maya, ladina/mestiza, garífuna, xinca o de otra etnia? | ¿Qué idioma o dialecto hablan habitualmente en su hogar? |
| **Survey question (English)*** | What languages ​​ do you speak? | According to your culture, people or physical characteristics, are you or do you recognize as: | How do you consider yourself: Mayan, Ladina/Mestizo, Garifuna, Xinca or another ethnic group? | What language or dialect do you usually speak at home? |
| **Survey response options (Spanish)** | Quechua (1) | Indígena (1) | Maya (1) | Castellano (1) |
|  | Aymara (2) | Gitano(a)/Rom (2) | Ladina/Mestiza (2) | Quechua (2) |
|  | Guaraní (3) | Raizal Del Archipiélago (3) | Garífuna (3) | Aymara (3) |
|  | Otro (6) | Palenquero(a) de San Basilio (4) | Xinca (4) | Otra lengua aborigen (4) |
|  | Ninguno (7) | Negro(a)/Mulato(a)/ Afrocolombiano(a)/Afrodescendiente (5) | Otro (6) | Idioma extranjero (5) |
|  |  | Ninguna de las anteriores (6) | No sabe/no está segura (8) |  |
| **Survey response options (English)*** | Quechua (1) | Indigenous (1) | Maya (1) | Spanish (1) |
|  | Aymara (2) | Gypsy/Rom (2) | Ladina/Mestiza (2) | Quechua (2) |
|  | Guarani (3) | Raizal of the Archipelago (3) | Garífuna (3) | Aymara (3) |
|  | Other (6) | Palenquero(a) of San Basilio (4) | Xinca (4) | Other Aboriginal language (4) |
|  | None (7) | Black/Mulatto(a)/Afro-Colombian/Afro-descendant (5) | Other (6) | Foreign language (5) |
|  |  | None of the above (6) | Don’t know/not sure (8) |  |
| Notes: * Author’s translation | | | | |

## Supplementary Figures

### Figure 1

**Supplementary Figure 1:** Workflow depicting data search, inclusion/exclusion of countries, waves, and samples

Demographic and Health Surveys data on 4 countries (1986–2015; N (level-1)=165,825; N (level-2)=5,055)

Latin American and Caribbean (LAC) countries

(N = 33)

LAC countries with no DHS data

(N = 18)

LAC countries after removing countries with no DHS data

(N = 15)*

LAC countries with ≤ 2 DHS data waves and/or collected only in the 1980s and 1990s

(N = 9)**

LAC countries after removing countries with ≤ 2 DHS data waves and/or waves collected only in the 1980s and 1990s

(N = 6)***

LAC countries with no DHS data on ethnoracial self-identification

(N = 2)****

LAC countries included in this analysis, after removing countries with no DHS data on ethnoracial self-identification

(N = 4)*****

**Bolivia**

Survey waves: 1989, 1994, 1998, 2003, 2008

N = 30,825

**Colombia**

Survey waves: 1986, 1990, 1995, 2000, 2005, 2010, 2015

N = 30,325

**Guatemala**

Survey waves: 1987, 1995, 1998–1999, 2014–2015

N = 16,966

**Peru**

Survey waves: 1986, 1991–1992, 1996, 2000, 2004–2006, 2007–2008, 2009, 2010, 2011, 2012

N = 87,709

Notes:

* Bolivia, Brazil, Colombia, Dominican Republic, Ecuador, El Salvador, Guatemala, Guyana, Haiti, Honduras, Mexico, Nicaragua, Paraguay, Peru, Trinidad and Tobago

** Brazil, Ecuador, El Salvador, Guyana, Honduras, Mexico, Nicaragua, Paraguay, Trinidad and Tobago

*** Bolivia, Colombia, Dominican Republic, Guatemala, Haiti, Peru

**** Dominican Republic, Haiti,

***** Bolivia, Colombia, Guatemala, Peru
